# [2,2′-Bis(pyridin-2-ylmeth­oxy)biphenyl-κ^4^ *N*,*O*,*O*′,*N*′]bis­(nitrato-κ^2^ *O*,*O*′)cadmium

**DOI:** 10.1107/S1600536811039687

**Published:** 2011-10-05

**Authors:** Cai-Juan Zhao, Rui-Feng Zhang

**Affiliations:** a School of Chemistry & Material Science, Shanxi Normal University, Linfen 041004, People’s Republic of China

## Abstract

In the title compound, [Cd(NO_3_)_2_(C_24_H_20_N_2_O_2_)], the Cd^II^ ion is eight-coordinated by one ligand and two nitrate ions. There are C—H⋯O hydrogen bonds and C—H⋯π inter­actions and π–π inter­actions [centroid–centroid distance = 3.319 (1) Å] in the crystal structure.

## Related literature

For background to weak inter­molecular inter­actions, see: Desiraju & Steiner (2001 ▶); Reinhoudt & Crego-Calama (2002 ▶); Frederik & Mikkel (2001 ▶). For the synthesis of the 2,2′-bis­(pyridin-2-ylmeth­oxy)biphenol ligand, see: Oh *et al.* (2005 ▶). For C—H⋯O and C—H⋯π hydrogen bonds, see: Guo *et al.* (2005 ▶). For aromatic ring arrangements, see: Janiak (2000 ▶). 
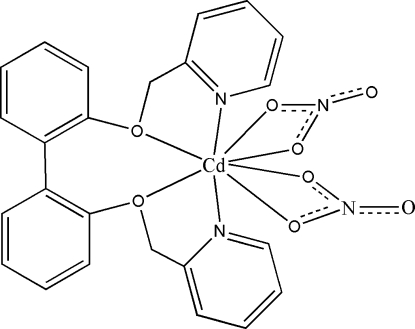


## Experimental

### 

#### Crystal data

[Cd(NO_3_)_2_(C_24_H_20_N_2_O_2_)]
*M* *_r_* = 604.84
Triclinic, 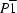

*a* = 9.2216 (14) Å
*b* = 10.1064 (16) Å
*c* = 14.335 (2) Å
α = 73.723 (2)°
β = 73.501 (2)°
γ = 75.199 (2)°
*V* = 1207.0 (3) Å^3^
*Z* = 2
Mo *K*α radiation
μ = 0.96 mm^−1^
*T* = 293 K
0.30 × 0.24 × 0.20 mm

#### Data collection

Bruker SMART CCD area-detector diffractometer
Absorption correction: multi-scan (*SADABS*; Sheldrick, 1996 ▶) *T*_min_ = 0.793, *T*_max_ = 1.000
6126 measured reflections
4197 independent reflections
3714 reflections with *I* > 2σ(*I*)
*R*_int_ = 0.017

#### Refinement

*R*[*F* ^2^ > 2σ(*F* ^2^)] = 0.025
*wR*(*F* ^2^) = 0.065
*S* = 1.05
4197 reflections
334 parameters
H-atom parameters constrained
Δρ_max_ = 0.35 e Å^−3^
Δρ_min_ = −0.45 e Å^−3^

### 

Data collection: *SMART-NT* (Bruker, 1998 ▶); cell refinement: *SAINT-NT* (Bruker, 1998 ▶); data reduction: *SAINT-NT*; program(s) used to solve structure: *SHELXS97* (Sheldrick, 2008 ▶); program(s) used to refine structure: *SHELXL97* (Sheldrick, 2008 ▶); molecular graphics: *XP* in *SHELXTL* (Sheldrick, 2008 ▶); software used to prepare material for publication: *SHELXTL*.

## Supplementary Material

Crystal structure: contains datablock(s) 060610a, I. DOI: 10.1107/S1600536811039687/jh2323sup1.cif

Structure factors: contains datablock(s) I. DOI: 10.1107/S1600536811039687/jh2323Isup2.hkl

Additional supplementary materials:  crystallographic information; 3D view; checkCIF report

## Figures and Tables

**Table 1:** Hydrogen-bond geometry (Å, °) *Cg*2 and *Cg*3 are the centroids of the N2/C20–C24 and C7–C12 rings, respectively.

*D*—H⋯*A*	*D*—H	H⋯*A*	*D*⋯*A*	*D*—H⋯*A*
C6—H6*B*⋯C4	0.97	2.67	3.278 (4)	121
C11—H11⋯O7^i^	0.93	2.46	3.119 (3)	128
C15—H15⋯O8^ii^	0.93	2.54	3.286 (4)	137
C19—H19*B*⋯O4^iii^	0.97	2.49	3.411 (4)	159
C1—H1⋯O5^iv^	0.93	2.58	3.317 (4)	136
C23—H23⋯O3^v^	0.93	2.64	3.330 (4)	131
C17—H17⋯*Cg*2^iii^	0.93	2.90	3.736 (3)	137
C22—H22⋯*Cg*3^vi^	0.93	2.90	3.693 (3)	144

Symmetry codes: (i) 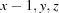; (ii) 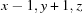; (iii) 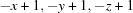; (iv) 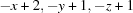; (v) 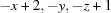; (vi) 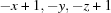.

**Fig. 1.**
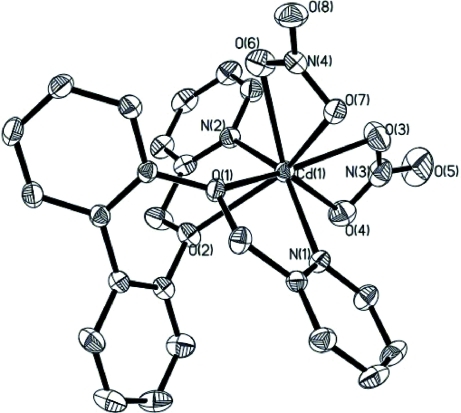
The thermal ellipsoid (30%) plot of coordination compound 1. Hydrogen atoms are omitted for clarity.

**Fig. 2.**
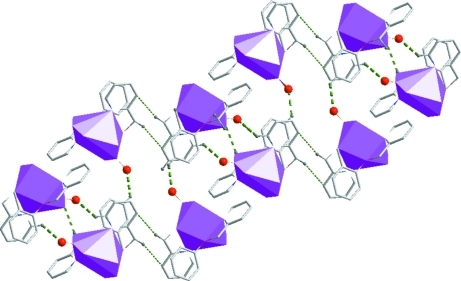
The hydrogen bonds of the title compound. Part of the hydrogen atoms are omitted for clarity.

**Fig. 3.**
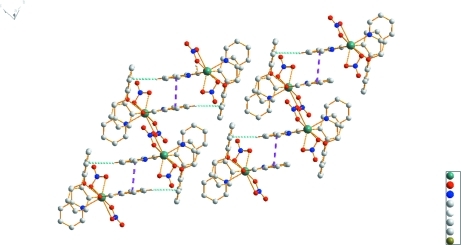
The π-π interactions of the title compound. Part of the hydrogen atoms are omitted for clarity.

## supplementary crystallographic information

### Comment

In the past few years, there has been increasing interest in the study of weak intermolecular interactions, because of their important roles played in the various fields of chemistry and biochemistry, such as crystal engineering, supramolecular chemistry, molecular recognition and self-assembly of molecules (Desiraju *et al.*, 2001). Utilization of supramolecular architecture *via* non-covalent interactions is a vigorous field involving in the creation of new functional materials and is a powerful tool for particular structure formations (Reinhoudt *et al.*, 2002). Within the field of supramolecular chemistry, the non-covalent linkage of *p*-electron donating molecules to a *p*-deficient acceptor moiety through hydrogen bond and/or cooperative aromatic interactions (Frederik *et al.*, 2001) has attracted much attention in recent years. Recently, we have synthesized a coordination compound, namely [Cd(*L*_1_)(NO_3_)_2_], (*L*_1_ = 2,2'-bis(pyridin-2-ylmethyl)biphenol), which is constructed through non-covalent interactions.

The title compound crystallizes in triclinic space group P-1. The asymmetrical unit of the unit cell contains one Cd(II) ion, one ligand *L*_1_ and two nitrate ions, as shown in Fig. 1. Each Cd(II) ion is eight-coordinated with four oxygen atoms from the two coordinated nitrate ions, two nitrogen and two oxygen atoms from the ligand *L*_1_. The compound is then constructed into three-dimensional supramolecular structure through hydrogen bonds and π-π interactions. There are two kinds of hydrogen bonds (as shown in Fig. 2), C—H···O (H···O 2.460 (5)–2.895 (0) Å, O···O 3.118 (9)–3.412 (0) Å) and C—H···π (H···C 2.895 (0) Å, C···C 3.843 (1) Å) respectively, which is in good agreement with values reported in literature (Guo *et al.*, 2005). Both face-to-face and edge-to-face aromatic rings arrangements exist in compound, as shown in Fig. 3. For the face-to-face arrangement, the dihedral angle is 0.186° and the centroid-centroid distance is 3.319 (1) Å, within the effective distance 3.3–3.8 Å (Janiak, 2000). As for the edge-to-face arrangement, the dihedral angle is 107.41° and H···cg' 2.900 (1) Å, C···cg' 3.693 (4) Å, C—H···cg' 144.04°, where C—H is from pyridine ring, and cg' is the centroid of the phenol ring.

### Experimental

The title compound was prepared by adding 5 ml methanol solution of cadmium nitrate (0.3 mmol) to 10 ml methanol solution of *L*_1_ (Oh *et al.*, 2005). The mixture was stirred for half an hour and filtered. The filtrate was slowly evaporated at room temperature to yield colorless block crystals suitable for X-ray analysis. Analysis calculated for C_24_H_20_CdN_4_O_8_: C 47.62, H 3.31, N 9.26%; found: C 48.06, H 3.09, N 9.49%.

### Refinement

Hydrogen atoms were included in calculated positions and refined with fixed thermal parameters riding on their parent atoms with C—H distances in the range of 0.93–0.97 Å, *U*_iso_(H) = 1.2*U*_eq_(C).

### Crystal data

**Table:**

[Cd(NO_3_)_2_(C_24_H_20_N_2_O_2_)]	*Z* = 2
*M**_r_* = 604.84	*F*(000) = 608
Triclinic, *P*1	*D*_x_ = 1.664 Mg m^−^^3^
*a* = 9.2216 (14) Å	Mo *K*α radiation, λ = 0.71073 Å
*b* = 10.1064 (16) Å	Cell parameters from 4044 reflections
*c* = 14.335 (2) Å	θ = 2.4–26.3°
α = 73.723 (2)°	µ = 0.96 mm^−^^1^
β = 73.501 (2)°	*T* = 293 K
γ = 75.199 (2)°	Block, colourless
*V* = 1207.0 (3) Å^3^	0.30 × 0.24 × 0.20 mm

### Data collection

**Table:**

Bruker SMART CCD area-detector diffractometer	4197 independent reflections
Radiation source: fine-focus sealed tube	3714 reflections with *I* > 2σ(*I*)
graphite	*R*_int_ = 0.017
φ and ω scans	θ_max_ = 25.0°, θ_min_ = 2.1°
Absorption correction: multi-scan (*SADABS*; Sheldrick, 1996)	*h* = −10→10
*T*_min_ = 0.793, *T*_max_ = 1.000	*k* = −10→12
6126 measured reflections	*l* = −17→13

### Refinement

**Table:**

Refinement on *F*^2^	Primary atom site location: structure-invariant direct methods
Least-squares matrix: full	Secondary atom site location: difference Fourier map
*R*[*F*^2^ > 2σ(*F*^2^)] = 0.025	Hydrogen site location: inferred from neighbouring sites
*wR*(*F*^2^) = 0.065	H-atom parameters constrained
*S* = 1.05	*w* = 1/[σ^2^(*F*_o_^2^) + (0.0345*P*)^2^ + 0.1926*P*] where *P* = (*F*_o_^2^ + 2*F*_c_^2^)/3
4197 reflections	(Δ/σ)_max_ = 0.001
334 parameters	Δρ_max_ = 0.35 e Å^−^^3^
0 restraints	Δρ_min_ = −0.45 e Å^−^^3^

### Special details

**Table:**

Geometry. All e.s.d.'s (except the e.s.d. in the dihedral angle between two l.s. planes) are estimated using the full covariance matrix. The cell e.s.d.'s are taken into account individually in the estimation of e.s.d.'s in distances, angles and torsion angles; correlations between e.s.d.'s in cell parameters are only used when they are defined by crystal symmetry. An approximate (isotropic) treatment of cell e.s.d.'s is used for estimating e.s.d.'s involving l.s. planes.
Refinement. Refinement of *F*^2^ against ALL reflections. The weighted *R*-factor *wR* and goodness of fit *S* are based on *F*^2^, conventional *R*-factors *R* are based on *F*, with *F* set to zero for negative *F*^2^. The threshold expression of *F*^2^ > σ(*F*^2^) is used only for calculating *R*-factors(gt) *etc*. and is not relevant to the choice of reflections for refinement. *R*-factors based on *F*^2^ are statistically about twice as large as those based on *F*, and *R*- factors based on ALL data will be even larger.

### Fractional atomic coordinates and isotropic or equivalent isotropic displacement parameters (Å^2^)

**Table:**

	*x*	*y*	*z*	*U*_iso_*/*U*_eq_	
Cd1	0.81711 (2)	0.308586 (19)	0.326615 (13)	0.03353 (8)	
O1	0.74199 (19)	0.35604 (18)	0.16450 (12)	0.0339 (4)	
O2	0.5313 (2)	0.4063 (2)	0.34234 (12)	0.0410 (4)	
O3	1.0172 (3)	0.2506 (3)	0.41079 (19)	0.0707 (7)	
O4	0.8298 (3)	0.3957 (2)	0.47524 (16)	0.0622 (6)	
O5	1.0254 (4)	0.3066 (4)	0.5432 (2)	0.1063 (11)	
O6	0.8992 (2)	0.0848 (2)	0.27615 (15)	0.0565 (5)	
O7	1.0435 (2)	0.2359 (2)	0.19232 (17)	0.0553 (5)	
O8	1.0681 (3)	0.0488 (2)	0.14034 (17)	0.0594 (6)	
N1	0.8489 (2)	0.5283 (2)	0.23165 (15)	0.0350 (5)	
N2	0.6624 (2)	0.1800 (2)	0.45773 (15)	0.0380 (5)	
N3	0.9588 (4)	0.3176 (3)	0.4785 (2)	0.0583 (7)	
N4	1.0062 (3)	0.1209 (2)	0.20242 (18)	0.0426 (6)	
C1	0.8922 (3)	0.6165 (3)	0.2697 (2)	0.0460 (7)	
H1	0.9034	0.5875	0.3353	0.055*	
C2	0.9203 (4)	0.7463 (3)	0.2163 (2)	0.0530 (8)	
H2	0.9484	0.8047	0.2453	0.064*	
C3	0.9061 (4)	0.7883 (3)	0.1194 (2)	0.0532 (8)	
H3	0.9267	0.8752	0.0810	0.064*	
C4	0.8610 (3)	0.7004 (3)	0.0791 (2)	0.0441 (7)	
H4	0.8495	0.7280	0.0136	0.053*	
C5	0.8331 (3)	0.5713 (2)	0.13708 (17)	0.0303 (5)	
C6	0.7855 (3)	0.4773 (3)	0.09140 (18)	0.0374 (6)	
H6A	0.6994	0.5291	0.0617	0.045*	
H6B	0.8703	0.4477	0.0388	0.045*	
C7	0.6315 (3)	0.2988 (3)	0.14698 (17)	0.0295 (5)	
C8	0.6794 (3)	0.1738 (3)	0.11518 (19)	0.0371 (6)	
H8	0.7830	0.1312	0.1035	0.044*	
C9	0.5704 (3)	0.1131 (3)	0.1011 (2)	0.0438 (7)	
H9	0.6005	0.0292	0.0798	0.053*	
C10	0.4169 (3)	0.1775 (3)	0.1188 (2)	0.0458 (7)	
H10	0.3439	0.1362	0.1101	0.055*	
C11	0.3717 (3)	0.3021 (3)	0.1490 (2)	0.0417 (6)	
H11	0.2682	0.3451	0.1593	0.050*	
C12	0.4783 (3)	0.3659 (3)	0.16473 (18)	0.0327 (5)	
C13	0.4303 (3)	0.5031 (3)	0.19484 (18)	0.0342 (6)	
C14	0.3582 (3)	0.6212 (3)	0.1351 (2)	0.0462 (7)	
H14	0.3376	0.6124	0.0775	0.055*	
C15	0.3169 (4)	0.7502 (3)	0.1589 (2)	0.0572 (8)	
H15	0.2674	0.8269	0.1184	0.069*	
C16	0.3488 (4)	0.7660 (3)	0.2427 (2)	0.0600 (9)	
H16	0.3225	0.8537	0.2582	0.072*	
C17	0.4200 (4)	0.6515 (3)	0.3039 (2)	0.0520 (8)	
H17	0.4408	0.6616	0.3610	0.062*	
C18	0.4598 (3)	0.5223 (3)	0.27970 (18)	0.0364 (6)	
C19	0.4273 (3)	0.3428 (3)	0.4250 (2)	0.0556 (8)	
H19A	0.3531	0.3146	0.4019	0.067*	
H19B	0.3715	0.4102	0.4656	0.067*	
C20	0.5102 (3)	0.2170 (3)	0.48684 (19)	0.0396 (6)	
C21	0.4245 (4)	0.1425 (3)	0.5720 (2)	0.0484 (7)	
H21	0.3181	0.1703	0.5904	0.058*	
C22	0.5004 (4)	0.0263 (3)	0.6289 (2)	0.0558 (8)	
H22	0.4457	−0.0255	0.6864	0.067*	
C23	0.6574 (4)	−0.0124 (3)	0.5998 (2)	0.0559 (8)	
H23	0.7106	−0.0901	0.6375	0.067*	
C24	0.7342 (4)	0.0652 (3)	0.5147 (2)	0.0483 (7)	
H24	0.8405	0.0381	0.4950	0.058*	

### Atomic displacement parameters (Å^2^)

**Table:**

	*U*^11^	*U*^22^	*U*^33^	*U*^12^	*U*^13^	*U*^23^
Cd1	0.03440 (12)	0.03312 (12)	0.03341 (12)	−0.00985 (8)	−0.00953 (8)	−0.00311 (8)
O1	0.0330 (9)	0.0346 (10)	0.0375 (10)	−0.0129 (8)	−0.0124 (7)	−0.0031 (8)
O2	0.0371 (10)	0.0485 (11)	0.0298 (9)	−0.0054 (8)	−0.0068 (8)	−0.0004 (8)
O3	0.0536 (14)	0.0862 (19)	0.0803 (17)	0.0044 (13)	−0.0283 (12)	−0.0340 (15)
O4	0.0686 (16)	0.0581 (14)	0.0603 (14)	−0.0142 (12)	−0.0097 (12)	−0.0170 (11)
O5	0.140 (3)	0.130 (3)	0.084 (2)	−0.043 (2)	−0.077 (2)	−0.0106 (18)
O6	0.0565 (13)	0.0527 (13)	0.0484 (12)	−0.0115 (10)	−0.0015 (10)	−0.0021 (10)
O7	0.0378 (11)	0.0477 (13)	0.0888 (16)	−0.0089 (9)	−0.0126 (10)	−0.0299 (11)
O8	0.0602 (14)	0.0467 (13)	0.0676 (14)	−0.0026 (10)	−0.0014 (11)	−0.0276 (11)
N1	0.0371 (12)	0.0332 (12)	0.0362 (12)	−0.0126 (9)	−0.0071 (9)	−0.0059 (9)
N2	0.0389 (13)	0.0362 (12)	0.0375 (12)	−0.0099 (10)	−0.0086 (10)	−0.0036 (10)
N3	0.070 (2)	0.0621 (18)	0.0519 (16)	−0.0240 (15)	−0.0273 (14)	−0.0038 (14)
N4	0.0340 (13)	0.0391 (14)	0.0552 (15)	−0.0001 (10)	−0.0154 (11)	−0.0122 (12)
C1	0.0556 (18)	0.0448 (17)	0.0446 (16)	−0.0167 (14)	−0.0133 (13)	−0.0126 (13)
C2	0.063 (2)	0.0402 (17)	0.064 (2)	−0.0217 (15)	−0.0096 (16)	−0.0191 (15)
C3	0.067 (2)	0.0307 (16)	0.060 (2)	−0.0194 (14)	−0.0097 (16)	−0.0023 (14)
C4	0.0483 (17)	0.0377 (16)	0.0425 (16)	−0.0106 (13)	−0.0087 (13)	−0.0026 (12)
C5	0.0261 (12)	0.0291 (13)	0.0325 (13)	−0.0054 (10)	−0.0027 (10)	−0.0058 (10)
C6	0.0378 (15)	0.0442 (16)	0.0306 (13)	−0.0171 (12)	−0.0050 (11)	−0.0038 (11)
C7	0.0332 (13)	0.0299 (13)	0.0278 (13)	−0.0101 (10)	−0.0100 (10)	−0.0036 (10)
C8	0.0358 (14)	0.0370 (15)	0.0383 (15)	−0.0048 (12)	−0.0111 (11)	−0.0077 (12)
C9	0.0568 (19)	0.0344 (15)	0.0475 (17)	−0.0107 (13)	−0.0202 (14)	−0.0103 (12)
C10	0.0505 (18)	0.0474 (17)	0.0498 (17)	−0.0223 (14)	−0.0206 (14)	−0.0064 (14)
C11	0.0369 (15)	0.0463 (17)	0.0456 (16)	−0.0109 (12)	−0.0163 (12)	−0.0064 (13)
C12	0.0342 (14)	0.0339 (14)	0.0310 (13)	−0.0074 (11)	−0.0117 (10)	−0.0039 (10)
C13	0.0300 (13)	0.0358 (14)	0.0357 (14)	−0.0041 (11)	−0.0077 (11)	−0.0083 (11)
C14	0.0464 (17)	0.0492 (18)	0.0416 (16)	0.0049 (14)	−0.0190 (13)	−0.0116 (13)
C15	0.067 (2)	0.0442 (18)	0.0501 (19)	0.0137 (15)	−0.0201 (16)	−0.0095 (14)
C16	0.080 (2)	0.0361 (17)	0.058 (2)	0.0022 (16)	−0.0112 (17)	−0.0180 (15)
C17	0.068 (2)	0.0483 (18)	0.0407 (16)	−0.0039 (15)	−0.0151 (14)	−0.0162 (14)
C18	0.0333 (14)	0.0407 (15)	0.0323 (14)	−0.0045 (11)	−0.0071 (11)	−0.0063 (11)
C19	0.0441 (17)	0.0459 (18)	0.0545 (19)	−0.0027 (14)	0.0040 (14)	0.0030 (14)
C20	0.0494 (17)	0.0372 (15)	0.0316 (14)	−0.0114 (13)	−0.0048 (12)	−0.0085 (11)
C21	0.0533 (18)	0.0525 (18)	0.0381 (16)	−0.0193 (15)	0.0021 (13)	−0.0126 (14)
C22	0.083 (2)	0.0518 (19)	0.0320 (16)	−0.0274 (17)	−0.0073 (15)	−0.0001 (13)
C23	0.080 (2)	0.0466 (18)	0.0416 (17)	−0.0201 (17)	−0.0256 (16)	0.0081 (14)
C24	0.0497 (17)	0.0452 (17)	0.0478 (17)	−0.0114 (14)	−0.0185 (14)	0.0025 (14)

### Geometric parameters (Å, °)

**Table:**

Cd1—N1	2.298 (2)	C7—C8	1.386 (3)
Cd1—N2	2.305 (2)	C7—C12	1.387 (3)
Cd1—O3	2.349 (2)	C8—C9	1.388 (4)
Cd1—O6	2.438 (2)	C8—H8	0.9300
Cd1—O1	2.4960 (16)	C9—C10	1.381 (4)
Cd1—O7	2.516 (2)	C9—H9	0.9300
Cd1—O2	2.5355 (18)	C10—C11	1.372 (4)
Cd1—O4	2.564 (2)	C10—H10	0.9300
O1—C7	1.402 (3)	C11—C12	1.402 (4)
O1—C6	1.433 (3)	C11—H11	0.9300
O2—C18	1.403 (3)	C12—C13	1.490 (4)
O2—C19	1.406 (3)	C13—C18	1.392 (4)
O3—N3	1.256 (3)	C13—C14	1.395 (4)
O4—N3	1.254 (3)	C14—C15	1.372 (4)
O5—N3	1.216 (3)	C14—H14	0.9300
O6—N4	1.265 (3)	C15—C16	1.373 (4)
O7—N4	1.253 (3)	C15—H15	0.9300
O8—N4	1.229 (3)	C16—C17	1.382 (4)
N1—C5	1.341 (3)	C16—H16	0.9300
N1—C1	1.353 (3)	C17—C18	1.377 (4)
N2—C20	1.331 (3)	C17—H17	0.9300
N2—C24	1.354 (3)	C19—C20	1.498 (4)
C1—C2	1.369 (4)	C19—H19A	0.9700
C1—H1	0.9300	C19—H19B	0.9700
C2—C3	1.368 (4)	C20—C21	1.387 (4)
C2—H2	0.9300	C21—C22	1.379 (4)
C3—C4	1.383 (4)	C21—H21	0.9300
C3—H3	0.9300	C22—C23	1.372 (5)
C4—C5	1.379 (4)	C22—H22	0.9300
C4—H4	0.9300	C23—C24	1.364 (4)
C5—C6	1.500 (3)	C23—H23	0.9300
C6—H6A	0.9700	C24—H24	0.9300
C6—H6B	0.9700		
			
N1—Cd1—N2	146.09 (8)	C4—C5—C6	118.2 (2)
N1—Cd1—O3	101.09 (9)	O1—C6—C5	111.3 (2)
N2—Cd1—O3	92.68 (9)	O1—C6—H6A	109.4
N1—Cd1—O6	128.60 (7)	C5—C6—H6A	109.4
N2—Cd1—O6	81.38 (7)	O1—C6—H6B	109.4
O3—Cd1—O6	90.97 (8)	C5—C6—H6B	109.4
N1—Cd1—O1	68.38 (6)	H6A—C6—H6B	108.0
N2—Cd1—O1	113.32 (7)	C8—C7—C12	122.3 (2)
O3—Cd1—O1	147.49 (7)	C8—C7—O1	118.4 (2)
O6—Cd1—O1	74.91 (6)	C12—C7—O1	119.4 (2)
N1—Cd1—O7	81.60 (7)	C7—C8—C9	119.0 (2)
N2—Cd1—O7	131.70 (7)	C7—C8—H8	120.5
O3—Cd1—O7	79.53 (8)	C9—C8—H8	120.5
O6—Cd1—O7	51.57 (7)	C10—C9—C8	119.9 (3)
O1—Cd1—O7	68.70 (6)	C10—C9—H9	120.1
N1—Cd1—O2	85.15 (7)	C8—C9—H9	120.1
N2—Cd1—O2	66.24 (7)	C11—C10—C9	120.4 (3)
O3—Cd1—O2	143.64 (7)	C11—C10—H10	119.8
O6—Cd1—O2	113.14 (7)	C9—C10—H10	119.8
O1—Cd1—O2	68.10 (5)	C10—C11—C12	121.3 (3)
O7—Cd1—O2	136.72 (6)	C10—C11—H11	119.3
N1—Cd1—O4	86.65 (7)	C12—C11—H11	119.3
N2—Cd1—O4	78.09 (7)	C7—C12—C11	117.1 (2)
O3—Cd1—O4	51.22 (8)	C7—C12—C13	121.3 (2)
O6—Cd1—O4	135.23 (7)	C11—C12—C13	121.5 (2)
O1—Cd1—O4	149.86 (7)	C18—C13—C14	116.9 (2)
O7—Cd1—O4	125.77 (7)	C18—C13—C12	122.8 (2)
O2—Cd1—O4	94.10 (7)	C14—C13—C12	120.2 (2)
C7—O1—C6	115.83 (18)	C15—C14—C13	121.8 (3)
C7—O1—Cd1	125.97 (13)	C15—C14—H14	119.1
C6—O1—Cd1	116.20 (14)	C13—C14—H14	119.1
C18—O2—C19	113.6 (2)	C14—C15—C16	120.0 (3)
C18—O2—Cd1	128.07 (15)	C14—C15—H15	120.0
C19—O2—Cd1	118.32 (16)	C16—C15—H15	120.0
N3—O3—Cd1	101.28 (18)	C15—C16—C17	120.0 (3)
N3—O4—Cd1	90.90 (17)	C15—C16—H16	120.0
N4—O6—Cd1	96.57 (16)	C17—C16—H16	120.0
N4—O7—Cd1	93.15 (16)	C18—C17—C16	119.6 (3)
C5—N1—C1	117.9 (2)	C18—C17—H17	120.2
C5—N1—Cd1	122.44 (16)	C16—C17—H17	120.2
C1—N1—Cd1	119.62 (18)	C17—C18—C13	121.8 (2)
C20—N2—C24	117.8 (2)	C17—C18—O2	119.3 (2)
C20—N2—Cd1	124.93 (18)	C13—C18—O2	118.9 (2)
C24—N2—Cd1	116.98 (18)	O2—C19—C20	110.9 (2)
O5—N3—O4	122.5 (3)	O2—C19—H19A	109.5
O5—N3—O3	121.1 (3)	C20—C19—H19A	109.5
O4—N3—O3	116.4 (3)	O2—C19—H19B	109.5
O8—N4—O7	120.7 (2)	C20—C19—H19B	109.5
O8—N4—O6	121.5 (2)	H19A—C19—H19B	108.0
O7—N4—O6	117.8 (2)	N2—C20—C21	122.5 (3)
N1—C1—C2	123.1 (3)	N2—C20—C19	119.1 (2)
N1—C1—H1	118.5	C21—C20—C19	118.4 (3)
C2—C1—H1	118.5	C22—C21—C20	118.6 (3)
C3—C2—C1	118.5 (3)	C22—C21—H21	120.7
C3—C2—H2	120.7	C20—C21—H21	120.7
C1—C2—H2	120.7	C23—C22—C21	119.4 (3)
C2—C3—C4	119.4 (3)	C23—C22—H22	120.3
C2—C3—H3	120.3	C21—C22—H22	120.3
C4—C3—H3	120.3	C24—C23—C22	118.9 (3)
C5—C4—C3	119.2 (3)	C24—C23—H23	120.6
C5—C4—H4	120.4	C22—C23—H23	120.6
C3—C4—H4	120.4	N2—C24—C23	122.9 (3)
N1—C5—C4	121.9 (2)	N2—C24—H24	118.5
N1—C5—C6	119.9 (2)	C23—C24—H24	118.5
			
N1—Cd1—O1—C7	−151.15 (19)	O3—Cd1—N2—C24	−30.7 (2)
N2—Cd1—O1—C7	−7.90 (19)	O6—Cd1—N2—C24	59.9 (2)
O3—Cd1—O1—C7	132.55 (19)	O1—Cd1—N2—C24	129.28 (19)
O6—Cd1—O1—C7	65.53 (18)	O7—Cd1—N2—C24	47.7 (2)
O7—Cd1—O1—C7	119.66 (18)	O2—Cd1—N2—C24	180.0 (2)
O2—Cd1—O1—C7	−57.64 (17)	O4—Cd1—N2—C24	−80.1 (2)
O4—Cd1—O1—C7	−115.03 (19)	Cd1—O4—N3—O5	−175.6 (3)
N1—Cd1—O1—C6	12.00 (16)	Cd1—O4—N3—O3	4.6 (3)
N2—Cd1—O1—C6	155.25 (16)	Cd1—O3—N3—O5	175.1 (3)
O3—Cd1—O1—C6	−64.3 (2)	Cd1—O3—N3—O4	−5.1 (3)
O6—Cd1—O1—C6	−131.32 (17)	Cd1—O7—N4—O8	167.7 (2)
O7—Cd1—O1—C6	−77.19 (16)	Cd1—O7—N4—O6	−9.6 (2)
O2—Cd1—O1—C6	105.51 (17)	Cd1—O6—N4—O8	−167.3 (2)
O4—Cd1—O1—C6	48.1 (2)	Cd1—O6—N4—O7	9.9 (2)
N1—Cd1—O2—C18	21.51 (19)	C5—N1—C1—C2	0.0 (4)
N2—Cd1—O2—C18	−177.1 (2)	Cd1—N1—C1—C2	176.9 (2)
O3—Cd1—O2—C18	123.6 (2)	N1—C1—C2—C3	−1.0 (5)
O6—Cd1—O2—C18	−108.6 (2)	C1—C2—C3—C4	1.4 (5)
O1—Cd1—O2—C18	−47.12 (19)	C2—C3—C4—C5	−0.9 (5)
O7—Cd1—O2—C18	−50.8 (2)	C1—N1—C5—C4	0.6 (4)
O4—Cd1—O2—C18	107.8 (2)	Cd1—N1—C5—C4	−176.28 (19)
N1—Cd1—O2—C19	−155.9 (2)	C1—N1—C5—C6	179.9 (2)
N2—Cd1—O2—C19	5.5 (2)	Cd1—N1—C5—C6	3.1 (3)
O3—Cd1—O2—C19	−53.8 (3)	C3—C4—C5—N1	−0.1 (4)
O6—Cd1—O2—C19	74.0 (2)	C3—C4—C5—C6	−179.5 (3)
O1—Cd1—O2—C19	135.5 (2)	C7—O1—C6—C5	150.6 (2)
O7—Cd1—O2—C19	131.8 (2)	Cd1—O1—C6—C5	−14.3 (3)
O4—Cd1—O2—C19	−69.6 (2)	N1—C5—C6—O1	8.1 (3)
N1—Cd1—O3—N3	79.4 (2)	C4—C5—C6—O1	−172.5 (2)
N2—Cd1—O3—N3	−69.5 (2)	C6—O1—C7—C8	106.3 (2)
O6—Cd1—O3—N3	−150.9 (2)	Cd1—O1—C7—C8	−90.5 (2)
O1—Cd1—O3—N3	146.34 (17)	C6—O1—C7—C12	−75.6 (3)
O7—Cd1—O3—N3	158.5 (2)	Cd1—O1—C7—C12	87.6 (2)
O2—Cd1—O3—N3	−17.6 (3)	C12—C7—C8—C9	−0.4 (4)
O4—Cd1—O3—N3	2.88 (17)	O1—C7—C8—C9	177.6 (2)
N1—Cd1—O4—N3	−109.93 (18)	C7—C8—C9—C10	0.0 (4)
N2—Cd1—O4—N3	100.52 (18)	C8—C9—C10—C11	0.8 (4)
O3—Cd1—O4—N3	−2.83 (17)	C9—C10—C11—C12	−1.3 (4)
O6—Cd1—O4—N3	36.0 (2)	C8—C7—C12—C11	−0.1 (4)
O1—Cd1—O4—N3	−143.23 (16)	O1—C7—C12—C11	−178.1 (2)
O7—Cd1—O4—N3	−32.8 (2)	C8—C7—C12—C13	−177.5 (2)
O2—Cd1—O4—N3	165.18 (17)	O1—C7—C12—C13	4.6 (3)
N1—Cd1—O6—N4	23.7 (2)	C10—C11—C12—C7	0.9 (4)
N2—Cd1—O6—N4	−173.92 (17)	C10—C11—C12—C13	178.3 (2)
O3—Cd1—O6—N4	−81.35 (16)	C7—C12—C13—C18	−58.0 (4)
O1—Cd1—O6—N4	68.99 (15)	C11—C12—C13—C18	124.7 (3)
O7—Cd1—O6—N4	−5.57 (14)	C7—C12—C13—C14	119.2 (3)
O2—Cd1—O6—N4	126.62 (15)	C11—C12—C13—C14	−58.0 (4)
O4—Cd1—O6—N4	−110.62 (16)	C18—C13—C14—C15	−0.5 (4)
N1—Cd1—O7—N4	−151.69 (16)	C12—C13—C14—C15	−177.9 (3)
N2—Cd1—O7—N4	21.10 (19)	C13—C14—C15—C16	1.1 (5)
O3—Cd1—O7—N4	105.31 (17)	C14—C15—C16—C17	−1.1 (5)
O6—Cd1—O7—N4	5.59 (14)	C15—C16—C17—C18	0.6 (5)
O1—Cd1—O7—N4	−81.70 (15)	C16—C17—C18—C13	0.0 (5)
O2—Cd1—O7—N4	−78.05 (18)	C16—C17—C18—O2	−179.7 (3)
O4—Cd1—O7—N4	128.63 (15)	C14—C13—C18—C17	0.0 (4)
N2—Cd1—N1—C5	−107.9 (2)	C12—C13—C18—C17	177.3 (3)
O3—Cd1—N1—C5	139.97 (19)	C14—C13—C18—O2	179.6 (2)
O6—Cd1—N1—C5	39.7 (2)	C12—C13—C18—O2	−3.0 (4)
O1—Cd1—N1—C5	−7.88 (17)	C19—O2—C18—C17	82.6 (3)
O7—Cd1—N1—C5	62.46 (19)	Cd1—O2—C18—C17	−94.9 (3)
O2—Cd1—N1—C5	−76.23 (19)	C19—O2—C18—C13	−97.0 (3)
O4—Cd1—N1—C5	−170.63 (19)	Cd1—O2—C18—C13	85.4 (3)
N2—Cd1—N1—C1	75.3 (2)	C18—O2—C19—C20	178.1 (2)
O3—Cd1—N1—C1	−36.8 (2)	Cd1—O2—C19—C20	−4.2 (3)
O6—Cd1—N1—C1	−137.1 (2)	C24—N2—C20—C21	−0.3 (4)
O1—Cd1—N1—C1	175.3 (2)	Cd1—N2—C20—C21	−173.7 (2)
O7—Cd1—N1—C1	−114.3 (2)	C24—N2—C20—C19	−179.5 (3)
O2—Cd1—N1—C1	107.0 (2)	Cd1—N2—C20—C19	7.0 (4)
O4—Cd1—N1—C1	12.6 (2)	O2—C19—C20—N2	−1.2 (4)
N1—Cd1—N2—C20	28.3 (3)	O2—C19—C20—C21	179.5 (3)
O3—Cd1—N2—C20	142.8 (2)	N2—C20—C21—C22	0.5 (4)
O6—Cd1—N2—C20	−126.6 (2)	C19—C20—C21—C22	179.7 (3)
O1—Cd1—N2—C20	−57.2 (2)	C20—C21—C22—C23	−0.1 (5)
O7—Cd1—N2—C20	−138.85 (19)	C21—C22—C23—C24	−0.4 (5)
O2—Cd1—N2—C20	−6.54 (19)	C20—N2—C24—C23	−0.3 (4)
O4—Cd1—N2—C20	93.4 (2)	Cd1—N2—C24—C23	173.7 (2)
N1—Cd1—N2—C24	−145.19 (19)	C22—C23—C24—N2	0.6 (5)

### Hydrogen-bond geometry (Å, °)

**Table:**

Cg2 and Cg3 are the centroids of the N2/C20–C24 and C7–C12 rings ,respectively.

**Table:**

*D*—H···*A*	*D*—H	H···*A*	*D*···*A*	*D*—H···*A*
C6—H6B···C4	0.97	2.67	3.278 (4)	121.
C11—H11···O7^i^	0.93	2.46	3.119 (3)	128.
C15—H15···O8^ii^	0.93	2.54	3.286 (4)	137.
C19—H19B···O4^iii^	0.97	2.49	3.411 (4)	159.
C1—H1···O5^iv^	0.93	2.58	3.317 (4)	136.
C23—H23···O3^v^	0.93	2.64	3.330 (4)	131.
C17—H17···Cg2^iii^	0.93	2.90	3.736 (3)	137
C22—H22···Cg3^vi^	0.93	2.90	3.693 (3)	144

Symmetry codes: (i) *x*−1, *y*, *z*; (ii) *x*−1, *y*+1, *z*; (iii) −*x*+1, −*y*+1, −*z*+1; (iv) −*x*+2, −*y*+1, −*z*+1; (v) −*x*+2, −*y*, −*z*+1; (vi) −*x*+1, −*y*, −*z*+1.
